# Contexts, affective and physical states and their variations during physical activity in older adults: an intensive longitudinal study with sensor-triggered event-based ecological momentary assessments

**DOI:** 10.1186/s12966-025-01724-9

**Published:** 2025-03-07

**Authors:** Julie Delobelle, Sofie Compernolle, Tomas Vetrovsky, Jelle Van Cauwenberg, Delfien Van Dyck

**Affiliations:** 1 Physical Activity & Health, Department of Movement and Sports Sciences, Ghent University, Watersportlaan 2, Ghent, 9000 Belgium; 2 Research Foundation Flanders (FWO), Brussels, Belgium; 3 Faculty of Physical Education and Sport, Charles University, Prague, Czech Republic; 4 Department of Public Health and Primary care, Ghent University, Ghent, Belgium; 5 School of Public Health, Université Libre de Bruxelles, Brussels, Belgium

**Keywords:** Ecological momentary assessment (EMA), Sensor-triggered event-based experience sampling, Affect, Physical and social context, Physical activity, Older adults

## Abstract

**Background:**

To design effective tailored interventions to promote physical activity (PA) among older adults, insights are needed into the contexts in which older adults engage in PA and their affective and physical experiences. Sensor-triggered event-based ecological momentary assessment (EMA) is an innovative method for capturing real-life contexts, as well as affective and physical states, during or immediately after specific events, such as PA. This study aimed to (1) describe the physical and social contexts, and the affective and physical states during PA among older adults, (2) evaluate how these constructs fluctuate during PA episodes, and (3) describe affective states during PA according to the context.

**Methods:**

An intensive longitudinal sensor-triggered event-based EMA study was conducted with 92 Belgian older adults (65 + years). During seven days, participants were monitored using a Fitbit, which triggered a smartphone-based questionnaire on the event-based EMA platform ‘HealthReact’ after a five-minute walk. Participants reported on contexts and affective (positive/negative valence) and physical states (pain and fatigue) during the PA event. Descriptive statistics and generalized mixed models were used for data analysis.

**Results:**

Older adults predominantly engaged in daily physical activities, such as walking for transport, leisure walking, and gardening, rather than structured exercise. They consistently reported high positive affect, low negative affect, and minimal physical complaints during PA. Furthermore, older adults mainly engage in physical activities alone, particularly in outdoor settings. Variations in contexts, affect, and fatigue were mostly driven by within-subject differences. The model showed significant differences across times of day, with negative affect being highest in the evening and fatigue lowest in the morning. Additionally, the physical and social context influenced negative affect (but not positive affect), with outdoor activities performed alone and indoor activities performed with others being associated with lower negative affect.

**Conclusions:**

While these findings could enhance the effectiveness of tailored PA interventions, it remains unclear whether the observed affective and physical states are causes or effects of PA, and whether the contexts in which the activities were performed align with older adults’ preferences. Further research is needed to explore these relationships and to better understand older adults’ preferred PA contexts.

**Supplementary Information:**

The online version contains supplementary material available at 10.1186/s12966-025-01724-9.

## Introduction

Performing regular physical activity (PA) is crucial for maintaining good health and preventing and managing chronic diseases such as cardiovascular disease, type 2 diabetes, stroke, and some types of cancer [1–3]. The World Health Organization (WHO) recommends that adults (18–65 years) and older adults (65+) should engage in at least 150 min of moderate-intensity aerobic PA or 75 min of vigorous-intensity aerobic PA per week, or a combination of both [4]. However, adhering to these guidelines tends to decrease with age [5], emphasizing the need to encourage an active lifestyle among older adults. In order to design effective interventions to promote PA among older adults, insights are needed into the physical and social contexts in which older adults engage in PA and their affective and physical experiences during PA.

Despite increasing research demonstrating the influence of both the physical [6, 7], and social environment [8] on PA, little is currently known about the specific contexts in which older adults engage in PA and how these contexts fluctuate and influence their affective and physical states. Furthermore, models based on hedonic motivation (emphasizing pursuit of pleasure and avoidance of pain) [9] and operant conditioning (associating behavior with rewards and punishments) [10] suggest that the affective experiences during and immediately after PA significantly influence future engagement in PA. Affect refers to the broad range of feelings people experience, encompassing both positive and negative states. Russell’s circumplex model of affect provides a theoretical framework for understanding affect along two primary dimensions: valence (ranging from positive to negative) and arousal (ranging from low to high energy) [11]. In this study, we focus on the valence dimension, which captures the degree to which an individual feels pleasant (e.g., happiness) or unpleasant (e.g., anxiety) in response to various contexts. Behaviors that generate desirable affective states, characterized by high levels of positive valence and low levels of negative valence, are more likely to be repeated in the future. In contrast, behaviors that are accompanied with negative affective valence states may discourage future behavior [12–14]. Additionally, laboratory research has already demonstrated the link between PA and affect [15, 16], but to date, little is known about the influence of context on this link. A systematic review of ecological momentary assessment studies (EMA) highlights that incidental PA (e.g., gardening, household tasks) at even low intensities often enhances affective well-being, particularly by boosting energy levels, while effects of PA intensity or duration appear inconsistent [17]. These discrepancies likely stem from contextual factors, such as environmental settings, weather conditions and social interactions, which significantly moderate the relationship between PA and affective well-being [17, 18]. In addition, negative physical complaints (e.g., pain and fatigue) are known to reduce older adults’ motivation for PA engagement [19]. However, current understanding of older adults’ affective and physical states during PA, how these fluctuate throughout the day and how they relate to the physical and social contexts in which older adults are physically active is limited.

Existing literature primarily relies on retrospective cross-sectional questionnaires [20], which have shown that activities typically occur in outdoor settings close to older adults’ residences, including venues like parks, shopping malls, and neighborhood streets [21] and that they prefer to engage in physical activities with their peers [22, 23], particularly with a partner [24]. Furthermore, previous research among older adults combining accelerometer data with GPS data, has shown that green environments resulted in greater moderate-to-vigorous PA [25] and a significant portion of daily PA is attributed to active commuting [26].

However, retrospective questionnaires are typically administered long after the activities occur, making it challenging for older adults to accurately recall and report about contexts and their affective and physical states during past activities, which may induce recall bias. Additionally, while GPS and accelerometer research can provide accurate data on location and PA, it does not capture affective and physical states during the activity. Furthermore, retrospective questionnaires that are administered only once cannot capture within-subject variations. Understanding these temporal fluctuations may provide valuable insights for developing health behavior change strategies, allowing for anticipation of real-time dynamics of determinants rather than viewing them as stable within individuals. EMA addresses this issue by collecting real-time data on contexts and affective states during activities. Previous EMA research using time-based prompts, where surveys are administered at fixed or random times throughout the day, has shown that constructs like fatigue are not stable and can fluctuate throughout the day [27]. However, in a time-based EMA study, individuals are prompted at fixed or randomly allocated times during the day [28]. This approach may result in missing many PA events, especially in populations where PA events are rare, such as in many older adult populations. Thus, the probability of prompting a questionnaire during a bout of PA is small leading to few opportunities to assess contexts and physical and affective states during older adults’ PA.

A promising method to examine within-subject variations and to perform simultaneous assessments of contexts, and physical and affective states during PA is sensor-triggered event-based EMA [28]. This method allows researchers to repeatedly capture real-time contexts and physical and affective states during or immediately after a specific event (e.g., short bout of PA), without inducing recall bias [29, 30].

Previous research using time-based EMA in adults (aged 25 years and older) already showed that affect during PA was related to the contexts in which the activity was performed [31]. Engaging in PA with other individuals resulted in higher levels of positive affect compared to solitary PA. For physical contexts, higher negative affect was reported when engaging in PA indoors as compared with outdoors [6, 31]. Thus, environments that evoke positive emotions may enhance PA promotion. In a study involving adults, the combination of walking-triggered electronic diaries and GPS data revealed significant variations in affective states influenced by social and physical environmental factors [32]. Despite these advancements, no research has described momentary affect in different physical and social contexts during older adults’ everyday lives.

The objectives of the current event-based EMA study were (1) to describe the contexts, affective (i.e., negative and positive affect) and physical states (i.e., pain and fatigue) of older adults during PA in a naturalistic setting as well as their variation within and between individuals, (2) to explore how these constructs vary over time (i.e., during PA episodes), and (3) to describe affect during PA according to the physical and social context. It is hypothesized that older adults primarily engage in PA in outdoor settings with peers. Contexts, affective and physical states are expected to show significant variability within PA episodes. Furthermore, positive affect during PA is anticipated to be higher in supportive social settings (e.g., engaging in activity with others) or pleasant physical contexts (outdoors in green spaces), whereas negative affect is expected to be lower in such environments.

## Methods

### Participants

Between March and October 2022, 92 healthy, community-dwelling older adults (65+), including those who reached the age of 65 during the year of the study, were recruited in Flanders, Belgium, through purposeful convenience sampling via organizations for older adults and social media. Purposeful sampling was employed to achieve a heterogeneous sample, considering socio-demographic factors such as age and gender. Participants were excluded from the study if they had been diagnosed with cognitive impairments (e.g., mild cognitive impairment, dementia) or were not able to walk at least 100 m independently. An equal distribution across gender and age groups was pursued.

### Procedures

An intensive longitudinal study was conducted using sensor-triggered event-based EMA during seven consecutive days. Prior to the monitoring period, participants were visited at home for a full explanation of the study protocol and completion of a socio-demographic and health status questionnaire. Participants were instructed to download the custom-made mobile application ‘HealthReact’ onto their smartphone and to consistently wear a Fitbit activity tracker (Inspire 2 or Ionic) on the non-dominant wrist. Those without a smartphone were provided with a Motorola G30 or Motorola E20 phone and offered a brief training to use these devices. Participants were asked to charge the Fitbit activity tracker and smartphone at night.

During seven days, participants received sensor-triggered event-based EMA surveys after each short bout of PA, defined as at least five minutes of continuous walking or running, with a maximum of six questionnaires per day. These questionnaires were delivered by the HealthReact mobile application (version 1.62) [33]. This application is designed for conducting time- and event-based EMA studies and enables researchers to automatically trigger an EMA survey after a predefined event of PA, as measured by Fitbit. The specific triggering criterion for the EMA survey was set at a minimum of 60 steps per minute for five consecutive minutes, ensuring a high sensitivity (approximately 99%) and specificity (approximately 98%) in accurately identifying periods of sustained walking [34]. However, since the total number of triggered surveys per participant was lower than expected (i.e., on average seven events per participant during the entire monitoring period) for the first twelve participants, we adapted the protocol and decided to allow one outlier (i.e., one minute with fewer than 60 steps) during the five-minute walk for the remaining participants. To prevent disruption of the ongoing walking bout, the EMA surveys were only dispatched after two minutes of physical inactivity, which was defined as less than 10 steps per minute. However, since Fitbit syncs with the Fitbit app only every 15 min, there may have been a delay between the completion of the walk and the detection of the event. To accommodate for this delay, the maximum time interval to detect a potential walking event in the past was set at 20 min. Furthermore, to obtain a proportional distribution of prompts across the day and to avoid overburdening participants within a short time frame, the minimum time interval between two prompts was set at 60 min.

Completing the EMA survey required approximately two minutes. To allow sufficient time for participants to respond, the EMA survey remained accessible for 30 min. Participants were reminded to complete the questionnaire every 10 min, and during the last 5 min before the EMA survey expired, they received reminders every minute. At the conclusion of the seven-day measurement period, Fitbit activity trackers and any provided smartphones were collected again. The study was reported according to the STROBE checklist (see Supplementary file 1). In addition, relevant aspects from the CREMAS checklist, such as latency, were also considered to address the specifics of EMA methodology [35].

### Description of materials

#### Baseline questionnaire

At baseline, each participant completed a paper-and-pencil questionnaire to assess socio-demographic and health-related variables, including age, gender, height, weight, educational level, marital status, number of (grand)children and pets and household situation (i.e., living alone or with others). Generic health status was assessed using the PROMIS-29 [36], which includes a Visual Analog Scale (VAS) score to assess pain. Considering the ongoing COVID-19 pandemic at the time of data collection, participants were also asked if the pandemic had affected their current PA levels and social interactions.

#### Sensor-triggered event-based EMA survey

In the sensor-triggered event-based EMA survey, participants’ physical activities, accompanying contexts, as well as their affective and physical states were evaluated. First, participants were asked to indicate whether or not they were active before the prompt was triggered. If the participants did not confirm being physically active before the trigger, the EMA survey was terminated. If the EMA survey continued, participants were asked about the type of activity they were performing, with answer categories including: (1) “leisure walking,” (2) “leisure biking,” (3) “walking for transport,” (4) “biking for transport,” (5) “household activities,” (6) “gardening,” (7) “shopping,” (8) “sport or exercise,” (9) “playing with grandchild,” or (10) “other.” If participants chose the answer category “other”, they were asked to specify what type of activity they were performing.

Next, physical and social contexts were assessed. Specifically, participants were asked whether they were indoors or outdoors during the PA event. Those who had been active indoors were asked to specify in which of the following indoor places they had been active: (1) “home,” (2) “shop,” (3) “home of relatives,” (4) “in a healthcare facility (e.g. with a GP or other),” (5) “in a public space (e.g., library, etc.),” or (6) “other.” For those who were active outdoors, answer categories included: (1) “in a natural environment,” (2) “in a built environment,” (3) “in a private environment (e.g., garden or terrace),” or (4) “other.” If participants responded “other”, they received the follow-up question, “Where specifically were you indoors/outdoors?”.

To gather information about the social context, participants were asked if they had engaged in PA alone or with others. For those who had been active with others, the following answer categories were provided: (1) “pets,” (2) “partner,” (3) “child(ren),” (4) “grandchild(ren),” (5) “other relatives,” (6) “friend(s),” (7) “treating physicians (e.g., GP, nurses, etc.),” (8) “neighbors,” (9) “acquaintances,” or (10) “other.” If participants chose the answer category “other”, specification was asked.

Next, participants’ affect during the PA event was examined using a dimensional approach, guided by Russell’s circumplex model of affect [11]. This framework conceptualizes affect along the dimension of valence, ranging from positive to negative. To capture these dimensions, we assessed enthusiasm and happiness as indicators of positive valence, and anxiety and nervousness as indicators of negative valence For instance, the participants were asked: “How enthusiastic were you while engaging in PA?”. Responses were rated on a 7-point Likert scale ranging from (0) “not at all” to (7) “very much.” These items were selected from the Experience Sampling Method (ESM) by Philippe Delespaul’s research team at the University of Maastricht [27, 37]. Finally, physical states experienced during the event were evaluated through assessments of pain and fatigue. For instance, the participants were asked “How much pain did you have while engaging in PA?”. Responses were recorded on a scale from (0) “not at all” to (7) “very much.”

All items were selected based on existing scientific literature [27, 38], and the results of a small-scaled elicitation study we conducted prior to the current event-based EMA study. Within this study, modal salient beliefs (i.e., the most commonly held beliefs about PA within the target group) were identified with six non-active older adults using qualitative interviews, and potential activities to be surveyed during the EMA study were explored using an activity diary [39, 40]. This way, all items were tailored as closely as possible to the target group.

### Data processing and statistical analysis

EMA survey responses, time stamps and Fitbit-recorded steps in minute epochs were extracted from HealthReact. To identify potential technical issues, time stamps of Fitbit syncs were also extracted. Participants without EMA data were not included in the analyses. Descriptive statistics were computed to summarize sample characteristics and daily steps.

In addition, metrics were calculated, including the number of surveys sent, the number of completed surveys, the number of surveys in which the behavior was confirmed through the validation question, and the incidence of technical issues encountered by participants. In addition, the true positive rate (i.e., the proportion of surveys that were truly triggered by walking events divided by the total triggered surveys that were answered) was calculated. In addition, the prompt latency (i.e., the time between the last sync and when participants began responding) was calculated. However, the version of HealthReact used in this study did not log the exact timestamp of when a survey was prompted but only recorded the time when participants began their responses. We approximated the prompt timing by identifying the timestamp of the device sync that occurred after the stepping event but before participants started responding to the EMA survey.

To reach the first objective, descriptive statistics were calculated to quantify the physical and social contexts and affective (i.e., negative and positive affect) and physical states (i.e., pain and fatigue) of older adults during PA. The positive affect score was computed as the mean of ‘enthusiasm’ and ‘happiness,’ while the score for negative affect was calculated as the mean of ‘nervousness’ and ‘anxiety.’ In EMA questions where participants ticked the ‘other’ category, their specifications were recoded using one of the provided answer categories whenever possible. In addition, intercept-only generalized mixed models were fitted using the lme4 package [41] to investigate the within- and between-subject variance of the constructs. These models take into account the hierarchical data structure of the data (i.e., three levels: repeated measurements nested within days, within individuals). Since the physical and social contexts were defined as a binary outcome variable (i.e., outdoors vs. indoors and alone vs. not alone, respectively), logistic mixed models were conducted to examine variations in physical and social context. Generalized linear mixed models were performed to investigate the variation in physical states (Poisson Log Hurdle Model) and affective states (Gamma Log Hurdle Models for negative affect, and Gamma Log Models for positive affect). Variance and link functions were selected based on Akaike’s Information Criterion (AIC). The selection of Hurdle Models was motivated by an overabundance of zero values in the outcome variables pain, fatigue, and negative affect. These variables were dichotomized as absent or present to run the first part of the Hurdle Models. To assess within-subject variability, participants that did not complete at least three EMA surveys (*N* = 8) were excluded from analysis. The between and within subjects variances of positive affect were estimated by calculating the intraclass correlation coefficient (ICC). In addition, given the non-normal distribution of the other outcome variables, levels of between and within variance of contexts, physical complaints and negative affect were calculated using the simulation approach [42]. The within-subject variance includes both within-day and between-day variance, as the simulation approach does not distinguish between these two components.

To achieve the second objective and investigate how contexts and physical and affective states fluctuate during PA episodes, time was included in the intercept-only models as an independent variable with three categories: times between 6 am and 11:59 am were considered as ‘morning’, between 12 pm and 5:59 pm as ‘afternoon’, and between 6 pm and 12 am as ‘evening’ (= reference category). When comparing morning to afternoon, afternoon served as the reference category. We assessed the models’ assumptions through visual examination of residual-versus-fit plots and normal probability plots of standardized residuals. An example of a linear mixed model with positive affect as dependent variable and time as categorical independent variable: positive_affect_gamma_log = glmer(posaffectR ~ timeR + (1|ID/Day), family = Gamma(link="log”), data = EMA_validated).

Finally, to reach the third objective, two generalized linear mixed models were run to describe affect during PA according to the physical and social context. We utilized a Gamma Log Hurdle Model for negative affect and a Gamma Log Model for positive affect. An interaction effect for the physical and social context was incorporated into the model, since it was hypothesized that the effects might be interdependent. Analyses were performed using R (version 4.3.1). P-values below 0.05 were considered statistically significant.

### Sample size

The sample size was determined to prevent overfitting rather than to achieve a specific power level [43]. In line with guidelines for regression models, a minimum of ten observations per predictor was considered sufficient [44]. For binary outcome models, based on observations from our study, where 19% of participants engaged in indoor PA and 37% engaged in PA with others, and accounting for an expected 80% compliance rate among older adults in EMA studies, we estimated that recruiting 66 participants would be adequate [45].

## Results

**Fig. 1:**
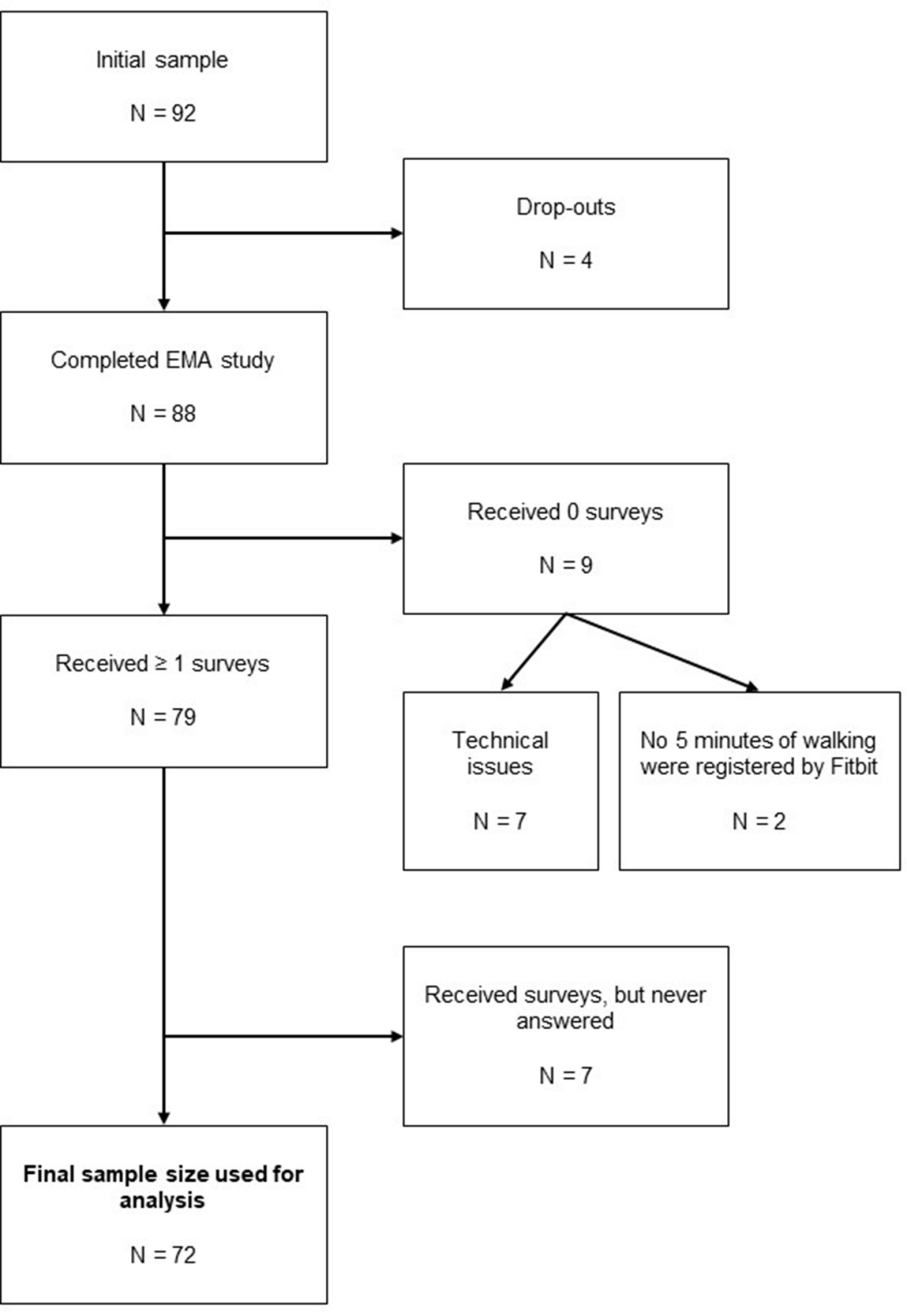
Flowchart of the sample size

Figure 1 provides a flowchart illustrating participant recruitment and exclusion. Initially, 92 individuals were recruited to participate in the study. However, four participants did not complete the one-week assessment period (e.g., because of experiencing too much stress due to study participation). Additionally, nine participants received no surveys at all, primarily due to synchronization problems between the Fitbit device and the Fitbit app. From the remaining 79 participants who received at least one survey, seven participants did not respond to any survey. This resulted in a final analytic sample of 72 participants, completing a total of 718 EMA surveys.

Table 1 presents the socio-demographic and health characteristics of the analytic sample. The median age was 71 (Q1 = 68, Q3 = 77) years and mean BMI was 26.3 ± 3.8 kg/m². Additionally, the sample included participants with a diverse range of educational backgrounds, relatively low pain scores, and high levels of PA (Table 1).

**Table 1:** Participant characteristics

Demographics	*N* = 72
Age (Mdn, Q1, Q3; range)	71, 68, 77; 64–90
Men (%)	54.2
BMI^a^ (M ± SD; range)	25.9 ± 3.7; 17.6–35.2
Education	
Elementary school (%)	15.3
Lower secondary (%)	29.2
Higher secondary (%)	20.8
Bachelor’s degree (%)	20.8
Master’s degree (%)	12.5
Doctoral degree (%)	1.4
Marital status	
Single (%)	2.8
Married or living together (%)	75.0
Divorced (%)	5.6
Widow/widower (%)	15.3
Having children (%)	93.1
Having grandchildren (%)	88.9
Living alone (%)	23.4
Physical activity affected by COVID (%)	29.6
Less physically active (%)	13.9
Social contact affect by COVID (%)	45.1
Less social contact (%)	43.7
VAS Pain 1–10 (Mdn, Q1, Q3; range)	2, 1, 3; 0–9
Daily steps across 7 days (Mdn, Q1, Q3; range)	8476, 5204, 12,473; 569-30424

*BMI*^*a*^: *body mass index (kg/m²)*,* Mdn: median*,* M: mean*,* SD: standard deviation*,* Q1: first quartile*,* Q3: third quartile*

In total 884 EMA surveys were sent, 718 were responded (compliance rate 81.2%) and 666 were confirmed as being triggered during/just after PA. On average, two EMA surveys (median = 2; Q1 = 1, Q3 = 3) were triggered and answered per participant per day, with one survey (median = 1; Q1 = 1, Q3 = 3) confirmed daily. A detailed overview of the number of triggered surveys during the monitoring period is provided in Table 2. The true positive rate (i.e., proportion of surveys that were truly triggered by walking events divided by the total triggered surveys that were answered) was 91.32%. This means that 8.68% of the triggers were misclassified, with prompts being issued without being preceded by five minutes of walking. Additionally, participants self-reported being physically active in 92.76% of the EMA surveys, and the median latency to answer the EMA survey was 3.13 min (IQR = 15.34).

**Table 2:** Triggered EMA surveys (*n* = 72)

	Per participant					
	Mdn	Q1-Q3	Range			
Triggered EMA surveys	11.0	7.0-14.50	2.0–34.0			
Answered EMA surveys	8.0	5.0-12.3	1.0–33.0			
Answered EMA surveys + confirmed PA	8.0	5.0–12.0	1.0–27.0			

Mdn: median, Q1: first quartile, Q3: third quartile

### Objective 1: describing the contexts, affective and physical states of older adults during PA

The descriptive statistics and variability in contexts, affective and physical states during PA are displayed in Table 3. The three primary types of physical activities were: (1) walking for transport (27.7%), (2) leisure walking (19.5%), and (3) gardening (18.4%). Additionally, the majority of PA events occurred alone (62.7%) and outdoors (81.1%). When participants engaged in physical activities with someone else, it was primarily with their partner (54.6%). The participants’ own homes emerged as the predominant indoor location for physical activities (56.0%). Considering outdoor PA, the majority of participants engaged in PA within built environments (39.9%), with fewer indicating natural settings (31.8%) or private environments (26.5%). The median values for negative affect and physical states were low (i.e., 0), whereas the median for positive affect was relatively high (i.e., 6). Furthermore, the majority of the variance in physical and social context, affect, and fatigue was explained by within-subject differences, unlike the variance in pain.

**Table 3:** Descriptive statistics and within and between subjects variance in contexts, affective and physical states during physical activity (*N* = 64)

EMA data	Descriptives (% or median (Q1- Q3))	Within subjects variance (%)	Between subjects variance (%)
**Activity**			
Walking for transport	27.7		
Leisure walking	19.5		
Gardening	18.4		
Biking for transport	8.0		
Leisure biking	6.9		
Household	5.5		
Sport or exercise	5.0		
Shopping	4.4		
Other	4.1		
Playing with grandchild(ren)	0.5		
**Social context**		67.5	32.5
Alone	62.7		
Not alone	37.3		
Partner	54.6		
Friends	22.7		
Pet	8.2		
Child(ren)	3.6		
Grandchild(ren)	2.7		
Other	2.7		
Acquaintance	2.3		
Other family members	1.8		
Neighbors	0.9		
Treating physician	0.5		
**Physical context (%)**		94.8	5.2
Indoors	18.9		
Home	56.0		
Shop	14.9		
Public open space	14.9		
Other	8.6		
Home of relatives	3.3		
Healthcare facility	2.3		
Outdoors	81.1		
Built environment	39.9		
Natural environment	31.8		
Private garden/terrace	26.5		
Other	1.8		
Affect*			
Negative	0 (0-0.5)	58.2	41.8
Positive	6 (4.5-7)	88.7	11.3
Physical states*			
Pain	0 (0–1)	48.9	51.1
Fatigue	0 (0–1)	65.1	34.9

^*^All items have a minimum of 0 and a maximum of 7,* ranging from “not at all” to “very much”*

### Objective 2: describing the fluctuation of contexts, affective and physical states

Supplementary file 2 gives an overview of the results of the generalized mixed models we conducted to investigate how contexts, physical and affective states fluctuate during PA episodes. First, a generalized logistic mixed model was generated to investigate the odds of engaging in PA with someone else and outdoors. The odds of engaging in PA with someone else were 60% (95% CI: 26–78%, *p* < 0.01) lower in the morning compared to in the afternoon. No significant differences in the odds of engaging in PA with someone else were found when comparing activities between morning and evening or between afternoon and evening. The odds of engaging in PA outdoors are on average 2.9 times higher in the morning (95% CI: 109–713%, *p* = 0.03) and 2.8 times higher in the afternoon (95% CI: 175–683%, *p* = 0.03) compared to the evening. No significant differences in odds of engaging in PA outdoors were found between morning and afternoon activities.

Furthermore, Supplementary file 2 presents the results of a Hurdle Model to investigate the fluctuation of negative affect during PA episodes at various times of the day. Time of the day does not influence the likelihood of experiencing negative affect during an event. However, among events during which negative affect was experienced, the level of negative affect was highest in the evening. More specifically, among events during which negative affect was experienced, those occurring in the morning and afternoon were associated with an average reduction in negative affect of 28% (95% CI: 13–41%, *p* < 0.01) and 33% (95% CI: 18–45%, *p* < 0.01), respectively, compared to events that occurred in the evening. There was no significant effect between morning and afternoon levels. Furthermore, no significant time fluctuations were found for positive affect.

Regarding the physical complaints, the Hurdle Model did not reveal significant fluctuations in pain and fatigue over time. However, among events during which fatigue was experienced, a trend towards significance was observed. More specifically, events occurring in the morning were associated with an average 16% (95% CI: -2-30%, *p* = 0.07) lower level of fatigue compared to events that occurred in the afternoon and 25% (95% CI: -2-45%, *p* = 0.07) lower level of fatigue compared to events that happened in the evening. There was no significant difference in level of fatigue between afternoon and evening activities.

### Objective 3: describing affect during PA according to the physical and social context

The third aim was to characterize the affective states during PA according to the physical and social context (see Table 4). Regarding the results for positive affect, the Gamma Log Model showed no significant interaction effect between the physical context and social context. Upon excluding the interaction effect, the Gamma Log Model showed no significant association between physical context (exp(b) = 1.27, 95% CI [0.93–1.13], *p* = 0.64) or social context (exp(b) = 1.06, 95% CI [0.98–1.14], *p* = 0.12) and positive affect.

**Table 4:** Overview of the generalized mixed models to describe affect during PA according to the physical and social context

	Gamma Log Model		Hurdle Model			
	Exp(b) with 95% CI	P-value	Logistic Model OR with 95% CI	P-value	Gamma Log Model Exp(b) with 95% CI	P-value
**Negative affect (ref. low score)**						
Social context (ref. alone)			1.77 (0.44–7.18)	0.42	0.77 (0.51–1.15)	0.20
Physical context (ref. indoors)			1.30 (0.56–3.03)	0.54	0.73 (0.56–0.94)	0.02
Social context*physical context			0.60 (0.14–2.65)	0.50	1.54 (1.02–2.34)	0.04*
**Positive affect**						
Social context (ref. alone)	1.21 (0.99–1.46)	0.05				
Physical context (ref. indoors)	1.07 (0.96–1.20)	0.23				
Social context*physical context	0.86 (0.70–1.06)	0.16				

*p-value < 0.05

For negative affect, the Logistic Hurdle Model showed no significant interaction effect between the physical and social context on the likelihood of experiencing negative affect. However, among those who experienced negative affect, a significant interaction effect was found in the Gamma Log Model between the physical and social context on the level of negative affect. This indicates that the relationship between negative affect and the social context significantly depends on the physical context. Activities performed alone are associated with lower levels of negative affect compared to when done with someone else, but only for outdoor activities. Conversely, this association is reversed for indoor activities; activities performed with others indoors are associated with lower levels of negative affect compared to activities performed alone.

## Discussion

This study aimed to (1) describe the physical and social contexts, and the affective and physical states during PA among older adults, (2) evaluate how these constructs fluctuate during PA episodes, and (3) describe affective states during PA according to the context.

### Main results

#### Physical and social contexts

This study found that nearly half of all detected PA events were performed in the form of walking, whether for leisure or commuting purposes. Besides walking, our results suggest that participants mainly perform activities that can be easily integrated into daily life, such as gardening and household chores. This observation aligns with previous research using traditional questionnaires [46, 47]. These activities are well-known for their health benefits in older adults and do not require planning or structuring as part of an exercise, as they occur naturally in daily life [47]. However, activities like these have been declining over the years due to technological innovations and digitalization, which reduce the need for physical effort in daily tasks (e.g., decreased active commuting, digital transformation of leisure activities.) [48]. Therefore, it is essential to continue promoting these daily activities as a simple but effective way to improve and maintain physical health and well-being, especially in an aging society.

In addition, our study revealed a predominant pattern of older adults engaging in physical activities alone. This pattern is inconsistent with existing literature indicating older adults’ preference for performing PA with others [23, 46]. Moreover, engaging in PA with others has been linked to improved mental well-being [49]. This may imply that while older adults may prefer to be physically active with others, they may not always have the opportunity to do so. However, our study also captured daily activities like gardening and household chores, which may be mainly performed alone and are often overlooked in current literature that predominantly concentrates on leisure PA [22, 23, 50]. Furthermore, traditional questionnaires might overlook these activities, as people typically associate PA with planned leisure activities. In addition, the sensor-triggered event-based EMA questionnaire was prompted already after a minimum of five minutes of sustained walking, making it plausible that shorter durations of PA are more likely to be pursued solitary. Such short bouts of PA may not have been captured in previous studies using traditional self-report questionnaires which often focus on activities lasting a minimum of ten minutes [51]. When activities were performed with someone else, it was usually with their partners which is in line with previous research [24, 52, 53].

Our results revealed that the majority of participants’ activities took place outdoors, with a significant portion occurring in built environments and natural environments. Indoor activities were predominantly conducted at home, underscoring the significance of both outdoor and home settings. This finding aligns with current literature, which highlights the importance of outdoor environments for PA and the role of the home environment in promoting regular activity, especially for older adults [54]. Interestingly, the study found that variability in physical and social contexts is largely due to within-subject differences. Differences in how, where, and with whom physical activities are performed vary significantly within the same individual across different times or contexts, rather than between different individuals.

Significant variations in these contexts throughout PA-episodes were observed, such as a decreased likelihood of engaging in PA with others during the morning compared to the afternoon. Morning routines are often centered around personal care, medical appointments, or solitary activities like walking or exercising alone to start the day [55]. By the afternoon, older adults might be more socially active, participating in group activities, community events, or meeting friends or family, leading to an increased likelihood of engaging in physical activities with others. In addition, the probability of participating in outdoor activities was lowest in the evening. This underrepresentation of evening outdoor activities may be due to higher perceived safety during daylight hours [56].

#### Affective and physical states

All participants consistently reported a relatively high level of positive affect and a low level of negative affect during PA events. Additionally, our study sample exhibited minimal physical complaints, such as pain and fatigue. This aligns with previous time-based EMA research in older adults, which found that high positive affect and low negative affect are associated with subsequent PA [57–59]. However, our findings primarily consist of descriptive results during bouts of PA; positive affect might have been high even before the activity began, or individuals experiencing negative affect may have chosen not to engage in PA at all. Therefore, it can be assumed that if individuals experience pain or negative affect, they might be unlikely to engage in PA. Furthermore, this sample might be considered a healthy sample of older adults, free from any physical complaints or other inconveniences, reducing the likelihood of individuals to report physical complaints.

We triggered EMA surveys after at least five minutes of walking to capture as many physical activity bouts as possible. However, laboratory research has shown that positive affect can emerge as early as three minutes into physical activity. The relationship between physical activity and affect is complex, influenced by factors such as intensity, duration, fitness levels, and psychological states [12, 60]. While positive affect may take time to emerge, moderate-intensity physical activity is generally associated with feelings of well-being, enjoyment, and energy both during and after activity [60–62]. Furthermore, laboratory research indicates that positive affect typically appears early—often within the first three minutes—and remains stable or increases as activity continues, unless prolonged fatigue sets in [63, 64]. In contrast, high-intensity physical activity is often linked to initial negative affective states, such as discomfort or exertion, with positive affect emerging after the activity is completed [15]. These timing of these affective responses also depend on baseline affective states, with individuals starting in a negative mood showing more pronounced improvement, and on fitness levels, as better-conditioned individuals tend to experience positive affect more quickly and at higher intensities [13, 65].

Interestingly, the current study revealed that the variability in positive as well as negative affect and fatigue during PA can be explained by within-subject differences. This means that fluctuations in an individual’s affect and levels of fatigue during PA are not solely due to differences between individuals, but are significantly influenced by personal variations in how each person experiences PA. However, these states were assessed exclusively during or immediately after PA bouts. Therefore, the observed within-subject variability in positive and negative affect might be lower than what might occur across an entire day. Incorporating mixed sampling schemes (such as combining event-based and time-based EMA) could enhance the interpretation by also assessing affect during non-PA moments. In addition, our findings indicate that the time of day is significantly associated with negative affect (but not with positive affect and fatigue). Specifically, negative affect was found to be highest during evening events. In addition, for events where fatigue was experienced, a trend towards significance indicated that morning events were associated with lower levels of fatigue compared to those in the afternoon and evening. However, the variation in pain could not be attributed to within-subject differences but rather to between-subject differences. This might be explained by the generally low levels of pain observed both during PA and in general. A more diverse and heterogeneous sample in terms of health status might be needed for exploring the variation of this construct.

Additionally, the context in which PA occurs was associated with negative affect, but not with positive affect. Specifically, outdoor activities performed alone were associated with lower levels of negative affect compared to activities done with others. This might be explained by the fact that solitude in outdoor settings may provide a sense of relief or relaxation [66], potentially reducing negative emotions more effectively than when engaging in these activities with others. Outdoor environments, which are often associated with exposure to natural elements and green spaces, are expected to have a positive impact on mood [31, 67–69]. Furthermore, a study combining walking-triggered electronic diaries and GPS data in adults, revealed significant associations between affective states (e.g., energetic arousal and calmness) and both social interactions and amount of greenness in the environment [32]. Participants reported lower energetic arousal when walking alone or in areas with less greenness, while walking with someone in greener settings was linked to higher levels of calmness [32]. These results align with our observation that levels of negative affect were lower during outdoor activities, suggesting a restorative potential of natural environments. Conversely, the pattern reverses for indoor activities. Here, engaging in activities with others indoors is associated with lower levels of negative affect compared to performing the same activities alone. This implies that the social context in indoors settings, may provide emotional support and reduce feelings of negative affect more effectively than in outdoor settings. Social interaction may provide emotional support and companionship, potentially enhancing mood and reducing feelings of loneliness [31, 70]. Finally, the relationship between positive affect and social context did not depend on the physical context in which activities occur. However, positive affect was generally high during PA, which may have limited potential for further enhancement through social interaction and an outdoor setting. Furthermore, positive affect encompasses a broad range of emotions [71], and the measurement instruments we employed to assess it might not have been sensitive enough to detect subtle differences.

### Recommendations for PA interventions and future research

In addition to the World Health Organization’ (WHO) PA guidelines, which recommend 150 min of moderate-intensity activity per week, the WHO also advises older adults to engage in muscle-strengthening, balance, and coordination exercises at least twice a week to help prevent falls [4]. The F.I.T.T. (Frequency, Intensity, Time, and Type) principles provide a framework for understanding and optimizing PA [72]. Frequency refers to how often PA is performed, intensity indicates the level of effort, time denotes the duration of activity, and type specifies the kind of activity. This framework is particularly useful for tailoring PA recommendations to individual needs and preferences. The predominance of walking and gardening as the most common types of activities in this study, suggests there may be potential to increase other types of physical activities, such as muscle-strengthening, balance, and coordination exercises. This could provide a valuable focus area for future interventions [73]. However, it’s important to note that the surveys in this study were triggered by stepping events, which primarily captured data on activities involving walking. This may have limited our ability to assess activities that do not involve stepping, such as muscle-strengthening exercises. Additionally, the median number of surveys triggered per day was low (i.e., one per day), suggesting that the frequency of PA events among older adults could be further improved. Regarding intensity, this study applied a threshold of at least 60 steps per minute to detect PA events. However, this threshold does not meet the benchmark for moderate-intensity PA, which is approximately 110 steps per minute. As a result, many captured events likely included light-intensity PA.

Future research could incorporate geographically-explicit ecological momentary assessment (GEMA) to address some of these limitations [74]. GEMA combines EMA methodologies with geolocation data, enabling a more precise understanding of how physical and social environments influence PA [75]. For example, using geolocation triggers, surveys could be tailored to capture activities occurring in specific contexts, such as gyms, parks, or homes, where non-stepping activities like muscle-strengthening or balance exercises might be more prevalent. GEMA could also identify patterns in environmental contexts, and how these factors interact with individual behaviors and affective and physical states [74]. Additionally, proximity detection within GEMA frameworks could assess the presence and quality of social interactions during PA episodes [76]. This integration could offer a more holistic view of how social and environmental contexts collectively impact both physical and affective states, paving the way for more targeted and effective interventions.

In addition, the sampling scheme employed in this study presents a limitation in capturing causal effects between PA and contextual, affective, and physical states. However, our objectives were explicitly of a descriptive nature. Future studies could investigate these causal effects by employing causal inference methods (e.g., directed acyclic graphs) and mixed sampling schemes, such as combining event-based and time-based EMA. This approach would enable the assessment of affective states before, during, and after PA, as well as during non-PA episodes,, providing a more comprehensive understanding of the dynamic interplay between PA, affective experiences, and contextual factors. Furthermore, by assessing these states exclusively during or immediately after PA bouts, we only captured the within-subject variability in positive and negative affect during PA episodes. The overall within-subject variability across an individual’s entire daily life may be higher than what was observed here, but this lies beyond the scope of the current paper.

In addition, the fluctuations in contexts throughout PA episodes suggest that older adults make deliberate choices about which physical activities they perform at different times, which should be taken into account when developing interventions aiming to promote PA among older adults [77]. However, it remains unclear whether these choices truly reflect older adults’ preferences. For example, older adults might actually prefer more opportunities for being physically active with others in the morning. Therefore, before developing interventions, it is essential to conduct qualitative research to understand whether the observed daily patterns align with the true preferences of older adults. Tailoring interventions to align with older adults’ variability in preferences has the potential to enhance the success of future efforts in motivating them to become or remain physically active. Just-In-Time Adaptive Interventions (JITAIs) are such a potential intervention strategy that can leverage contextual information to send encouraging prompts at the right time and context [78]. Consequently, JITAIs could suggest activities in the afternoon that can be performed with others. In addition, since the likelihood of engaging in PA outdoors is higher in the morning and afternoon compared to in the evening, individuals could be encouraged to spend time outdoors during daytime. Furthermore, strategies could be explored to promote PA outdoors in the evening. Finally, the current study revealed that fatigue was lowest during PA events happening in the morning. Prompts or reminders for PA could be sent during the morning, when fatigue levels are lowest, to capitalize on the individual’s higher energy levels and likelihood of engagement. This could involve sending motivational messages or providing suggestions for morning exercise routines tailored to the individual’s preferences and capabilities.

### Limitations and strengths

This study entails several limitations. First, our study sample was recruited through purposeful convenience sampling, which may have restricted the generalizability of our findings. Additionally, the sample included relatively active older adults with a slightly lower BMI (25.9) compared to the general population in Belgium (26.3), potentially resulting in lower levels of pain and fatigue [79]. Similarly, the average daily step count in our sample (8476 steps) was notably higher than what is typically observed among the general older adult population in Belgium, where only 20.5% (95% CI: 18.1–22.9) meet the PA guidelines. These differences suggest that our participants may represent a healthier and more active subset of the older adult population, potentially influencing the observed associations and limiting the applicability of our findings to less active or more diverse populations. Second, technical challenges, including synchronization issues between Fitbit and HealthReact, as well as a limited familiarity with smartphones among the participants, may have resulted in the omission of certain PA events. For example, seven participants who completed the seven-day monitoring period did not receive any sensor-triggered event-based EMA prompts due to technical issues. This may potentially have led to an underrepresentation of events and their associated contexts and affective and physical states in our study. In addition, participants who provided a higher number of responses may have a disproportionate influence on the results compared to those who completed only a few EMA surveys. To maximize inclusivity, smartphones were provided to participants who did not own one. However, a recently published paper including the data of the current study, showed that those without their own smartphones exhibited lower compliance rates [45]. Third, the definition of an event in terms of steps inevitably excludes other forms of PA, such as cycling, swimming and strength or flexibility exercises. Although Fitbit does not convert cycling to steps [80], our observations revealed instances where Fitbit registered steps during bike riding, potentially attributed to repetitive wrist movements or uneven terrains. This phenomenon contributes to the underrepresentation of cycling compared to walking as a reported activity. In addition, strength training typically involves stationary movements that do not generate the required number of steps to trigger the EMA survey. Despite thorough protocol testing, it is important to recognize that our technological choices may have resulted in missed events. These challenges highlight the need for clear guidelines regarding methodological aspects, including sampling type, prompt frequency, monitoring period, devices and EMA platforms, as well as technology choices such as start and end rules when implementing sensor-triggered event-based sampling. Fourth, participants were given a 30-minute window to allow sufficient time to complete the EMA questionnaire. However, it is important to acknowledge that this extended time frame may have introduced limited recall bias in participant responses. Fifth, it is important to acknowledge that some participants reported still being affected by COVID-19 during data collection. However, data collection spanned from March to October 2022 and all restrictions were dropped on March 7, 2022. Sixth, the primary objective was to describe contexts, affective and physical states during PA. It was not our purpose to examine causal effects of contexts on affect. Future research using intensive longitudinal data combined with causal inference methods (e.g., directed acyclic graphs) are needed to elucidate causal relationships between these constructs [81]. Finally, participants were not explicitly screened for mental health disorders beyond cognitive impairments. Since mental health conditions such as depression and anxiety can influence both PA patterns and affective states, future studies should consider including mental health assessments to better understand their potential moderating effects.

Despite its limitations, this study also encompasses several strengths. First, this study employs sensor-triggered event-based EMA, an innovative methodology in PA research. This approach enables researchers to repeatedly capture experiences, emotions and contexts during or immediately after a specific event (e.g., short bout of PA) in real-time. This design enabled us to capture short during physical activities, which are often overlooked while using traditional questionnaires [82, 83]. Second, this is one of the first non-self-initiated event-based EMA studies conducted among older adults. Despite potential concerns about the feasibility of conducting mobile-based EMA studies in older adults with no prior experience with smartphones [45], satisfactory response rates of 82.2% were observed in the current study. While technical issues led to some participant loss and missing data, emphasizing the importance of thorough smartphone usage instructions, our results align with prior studies demonstrating the viability of EMA in older populations [27, 84–86]. Thirdly, as data collection spanned from March to October 2022, our study captured information across diverse weather conditions. This extended time frame enhances the generalizability of our findings, albeit with the exception of the winter season.

## Conclusion

In conclusion, activities that can be easily integrated into older adults’ daily life, such as walking for transport, gardening, and household chores, are most common. Furthermore, we found that they predominantly engage in physical activities alone, particularly in outdoor settings, which may contradict existing literature suggesting a preference for social interaction during PA. This discrepancy suggests a possible gap between opportunities and preferences, warranting further qualitative research to explore whether current activity patterns align with older adults’ true preferences. Moreover, a substantial portion of the variability in affect, fatigue, and the physical and social context is driven by within subject variance. The study also highlights the association of context and time of day on affective states during PA, with negative affect during PA peaking in the evening and fatigue being lowest in the morning. These insights could enhance the effectiveness of PA interventions by enabling the development of real-time, context-sensitive support strategies tailored to an individual’s current state and context. However, the descriptive nature of this study limits the ability to draw causal conclusions and offers no insight into the direction of the relationship between these constructs and PA. Future sensor-triggered event-based EMA studies should collect data both during active and inactive episodes, incorporating a within-person encouragement design [87]. This design involves randomly assigning EMA prompts within participants that include varying levels of encouragement (e.g., prompts encouraging walking, or sitting down). By applying this approach, future research can gain a more comprehensive understanding of the causal relationships between PA and contexts, affective and physical states.

## Electronic supplementary material

Below is the link to the electronic supplementary material.

Supplementary Material 1

Supplementary Material 2

## Data Availability

The dataset supporting the conclusions of this article is available in the Open Science Framework repository, [https://osf.io/94tcb/].

## Abbreviations

EMA: Ecological momentary assessment
BMI: Body mass index
SD: Standard deviation
JITAI: Just-in-time-adaptive interventions
OR: Odds ratio
CI: Confidence interval
