# Supplementary material for: Contexts, affective and physical states and their variations during physical activity in older adults: an intensive longitudinal study with sensor-triggered event-based ecological momentary assessments
Source: Int J Behav Nutr Phys Act. 2025 Mar 7;22:30. doi: 10.1186/s12966-025-01724-9 (PMC11889861; doi:10.1186/s12966-025-01724-9)
Supplement: Supplementary file 1 — Supplementary Material 1 [file 12966_2025_1724_MOESM1_ESM.doc]

STROBE Statement—Checklist of items that should be included in reports of ***cross-sectional studies***

|  | Item No | Recommendation |
| --- | --- | --- |
| **Title and abstract** | 1 | (*a*) Indicate the study’s design with a commonly used term in the title or the abstract Study design is mentioned in the abstract and title. |
|  |  | (*b*) Provide in the abstract an informative and balanced summary of what was done and what was found Ok, see abstract |
| Introduction | | |
| Background/rationale | 2 | Explain the scientific background and rationale for the investigation being reported Ok, see Introduction. |
| Objectives | 3 | State specific objectives, including any prespecified hypotheses Objectives are clearly stated at the end of the introduction (page 4). We did not have prespecified hypotheses to test in this study, so these were not included. |
| Methods | | |
| Study design | 4 | Present key elements of study design early in the paper The study design is presented in the second paragraph of the Methods section. |
| Setting | 5 | Describe the setting, locations, and relevant dates, including periods of recruitment, exposure, follow-up, and data collection These elements are all explained in the ‘Participants’ and ‘Procedure’ paragraph in the Methods section. |
| Participants | 6 | (*a*) Give the eligibility criteria, and the sources and methods of selection of participants This is explained in the subsection ‘Participants’, pages 4-5 of the manuscript. |
| Variables | 7 | Clearly define all outcomes, exposures, predictors, potential confounders, and effect modifiers. Give diagnostic criteria, if applicable This was thoroughly explained in the Methods section, subtitle ‘Data processing and statistical analysis’. |
| Data sources/ measurement | 8* | For each variable of interest, give sources of data and details of methods of assessment (measurement). Describe comparability of assessment methods if there is more than one group All necessary information is given in the subsection ‘Description of materials’ on pages 6, 7 and 8. |
| Bias | 9 | Describe any efforts to address potential sources of bias This was explained in the subsection ‘Data processing and analysis’. |
| Study size | 10 | Explain how the study size was arrived at This was explained in the Methods section (Sample size). |
| Quantitative variables | 11 | Explain how quantitative variables were handled in the analyses. If applicable, describe which groupings were chosen and why See ‘Data processing and analysis’, page 8 and 9. |
| Statistical methods | 12 | (*a*) Describe all statistical methods, including those used to control for confounding |
|  |  | (*b*) Describe any methods used to examine subgroups and interactions |
|  |  | (*c*) Explain how missing data were addressed |
|  |  | (*d*) If applicable, describe analytical methods taking account of sampling strategy |
|  |  | (*e*) Describe any sensitivity analyses  A, b, c, d and e are all thoroughly explained in the ‘Data processing and analysis’-section, pages 8-9 of the manuscript. |
| Results | | |
| Participants | 13* | (a) Report numbers of individuals at each stage of study—eg numbers potentially eligible, examined for eligibility, confirmed eligible, included in the study, completing follow-up, and analysed  These numbers are provided in the first paragraph of the Results section. |
|  |  | (b) Give reasons for non-participation at each stage See page 10. |
|  |  | (c) Consider use of a flow diagram A flow diagram is presented at page 11 of the manuscript (Figure 1). |
| Descriptive data | 14* | (a) Give characteristics of study participants (eg demographic, clinical, social) and information on exposures and potential confounders See Table 1 for characteristics of the study participants. Information regarding confounders is provided in the Data processing and analysis section. |
|  |  | (b) Indicate number of participants with missing data for each variable of interest Participants without EMA data were excluded from the analyses. A flowchart illustrating the sample size, including the exclusion of participants without valid EMA data, is provided in Figure 1. Furthermore, details regarding the number of surveys sent to participants and their corresponding response rates are presented in the manuscript text (see page 14) and summarized in Table 2. |
| Outcome data | 15* | Report numbers of outcome events or summary measures See Table 2 and 3. |
| Main results | 16 | (*a*) Give unadjusted estimates and, if applicable, confounder-adjusted estimates and their precision (eg, 95% confidence interval). Make clear which confounders were adjusted for and why they were included See Tables 4, Additional file 2 and Data processing and analysis section. |
|  |  | (*b*) Report category boundaries when continuous variables were categorized See Data processing analysis section (page 8-9 of the manuscript). |
|  |  | (*c*) If relevant, consider translating estimates of relative risk into absolute risk for a meaningful time period This is not relevant for our manuscript |
| Other analyses | 17 | Report other analyses done—eg analyses of subgroups and interactions, and sensitivity analyses All analyses conducted are explained in the Data processing and analysis section. |
| Discussion | | |
| Key results | 18 | Summarise key results with reference to study objectives The key findings are summarized in the first paragraph of the Discussion section (subsection Physical and Social Contexts, page 17) and in the first paragraph of the Physical and Affective States subsection (pages 18-19). |
| Limitations | 19 | Discuss limitations of the study, taking into account sources of potential bias or imprecision. Discuss both direction and magnitude of any potential bias Limitations of the study were discussed on pages 21-22 of the manuscript. |
| Interpretation | 20 | Give a cautious overall interpretation of results considering objectives, limitations, multiplicity of analyses, results from similar studies, and other relevant evidence This was done in the Discussion section of the paper (pages 17 to 23 in the manuscript. |
| Generalisability | 21 | Discuss the generalisability (external validity) of the study results The generalisability is discussed in the Limitations and strengths section (pages 21-22) of the manuscript. |
| Other information | | |
| Funding | 22 | Give the source of funding and the role of the funders for the present study and, if applicable, for the original study on which the present article is based Funding information is given under Declarations. |

*Give information separately for exposed and unexposed groups.

**Note:** An Explanation and Elaboration article discusses each checklist item and gives methodological background and published examples of transparent reporting. The STROBE checklist is best used in conjunction with this article (freely available on the Web sites of PLoS Medicine at http://www.plosmedicine.org/, Annals of Internal Medicine at http://www.annals.org/, and Epidemiology at http://www.epidem.com/). Information on the STROBE Initiative is available at www.strobe-statement.org.
