# Supplementary material for: Contexts, affective and physical states and their variations during physical activity in older adults: an intensive longitudinal study with sensor-triggered event-based ecological momentary assessments
Source: Int J Behav Nutr Phys Act. 2025 Mar 7;22:30. doi: 10.1186/s12966-025-01724-9 (PMC11889861; doi:10.1186/s12966-025-01724-9)
Supplement: Supplementary file 2 — Supplementary Material 2 [file 12966_2025_1724_MOESM2_ESM.docx]

**Additional file 2.** Overview of the results of the generalized mixed models to investigate the fluctuation of contexts, affective and physical states within days.

|  | **Gamma Log Model** | | **Logistic Model** | | **Hurdle Model** | | | |
| --- | --- | --- | --- | --- | --- | --- | --- | --- |
|  | Exp(b) with 95% CI | P-value | OR with 95% CI | P-value | Logistic Model  OR with 95% CI | P-value | Gamma/Poisson Log Model  Exp(b) with 95% CI | P-value |
| **Social context (ref. alone)** |  |  |  |  |  |  |  |  |
| Morning vs afternoon^a^ |  |  | 0.40 (0.22 – 0.74) | <0.01** |  |  |  |  |
| Morning vs evening^a^ |  |  | 0.53 (0.16 – 1.73) | 0.30 |  |  |  |  |
| Afternoon vs evening^a^ |  |  | 1.33 (0.42 – 4.24) | 0.63 |  |  |  |  |
| **Physical context (ref. indoors)** |  |  |  |  |  |  |  |  |
| Morning vs afternoon^a^ |  |  | 1.03 (0.59 – 1.80) | 0.90 |  |  |  |  |
| Morning vs evening^a^ |  |  | 2.89 (1.09 – 7.13) | 0.03* |  |  |  |  |
| Afternoon vs evening^a^ |  |  | 2.79 (1.75 – 6.83) | 0.03* |  |  |  |  |
| **Negative affect (ref. low score)** |  |  |  |  |  |  |  |  |
| Morning vs afternoon^a^ |  |  |  |  | 1.11 (0.69 – 1.80) | 0.65 | 1.06 (0.93 – 1.21) | 0.38 |
| Morning vs evening^a^ |  |  |  |  | 0.60 (0.25 – 1.47) | 0.26 | 0.72 (0.59 – 0.87) | <0.01** |
| Afternoon vs evening^a^ |  |  |  |  | 0.54 (0.22 – 1.30) | 0.17 | 0.67 (0.55 – 0.82) | <0.01** |
| **Positive affect** |  |  |  |  |  |  |  |  |
| Morning vs afternoon^a^ | 1.02 (0.95 – 1.09) | 0.55 |  |  |  |  |  |  |
| Morning vs evening^a^ | 1.06 (0.93 – 1.21) | 0.39 |  |  |  |  |  |  |
| Afternoon vs evening^a^ | 1.04 (0.91 – 1.18) | 0.58 |  |  |  |  |  |  |
| **Pain (ref. low score)** |  |  |  |  |  |  |  |  |
| Morning vs afternoon^a^ |  |  |  |  | 1.09 (0.66 – 1.80) | 0.73 | 0.88 (0.72 – 1.09) | 0.25 |
| Morning vs evening^a^ |  |  |  |  | 0.98 (0.38 – 2.57) | 0.97 | 0.76 (0.54 – 1.07) | 0.11 |
| Afternoon vs evening^a^ |  |  |  |  | 0.90 (0.35 – 2.32) | 0.82 | 0.86 (0.61 – 1.20) | 0.37 |
| **Fatigue (ref. low score)** |  |  |  |  |  |  |  |  |
| Morning vs afternoon^a^ |  |  |  |  | 0.90 (0.58 – 1.38) | 0.62 | 0.84 (0.70 – 1.01) | 0.07 |
| Morning vs evening^a^ |  |  |  |  | 0.79 (0.34 – 1.86) | 0.59 | 0.75 (0.55 – 1.02) | 0.07 |
| Afternoon vs evening^a^ |  |  |  |  | 0.88 (0.38 – 2.07) | 0.77 | 0.89 (0.66 – 1.20) | 0.44 |

^a^Reference category was afternoon or evening. For the comparison between afternoon and evening, evening was set as the reference category.

*p-value < 0.05, **p-value < 0.01
